# ox-LDL-Induced Endothelial Progenitor Cell Oxidative Stress via p38/Keap1/Nrf2 Pathway

**DOI:** 10.1155/2022/5897194

**Published:** 2022-01-31

**Authors:** Qijun Jiang, Qiao Chen, Chengpeng Li, Zhigang Gong, Zhigang Li, Shifang Ding

**Affiliations:** ^1^Department of Cardiology, Affiliated Liyuan Hospital, Tongji Medical College, Huazhong University of Science and Technology, Yanhu Road, 39 Wuhan, Hubei Province, China; ^2^Department of Cardiology, General Hospital of Central Theater Command, Wuluo Road 627, Wuhan, 430070 Hubei Province, China

## Abstract

**Background:**

Nrf2 which was recently reported to regulate the antioxidant genes and cellular redox regulators was highly expressed in EPCs. However, its role in ox-LDL-induced EPC oxidative stress and apoptosis has not been fully illustrated.

**Methods:**

EPCs isolated from human peripheral blood mononuclear cells were treated with different concentrations of ox-LDL, Keap1 siRNA, and a specific p38 MAPK inhibitor SB203580 and then used to assay the cytoplasmic Nrf2, nuclear Nrf2, NAD(P) H:quinone oxidoreductase 1 (NQO1) and Bax/Bcl-2 levels with Western blot, NQO1 mRNA levels with RT-PCR, ROS levels with H2DCF-DA, loss/disruption of mitochondrial membrane potential with JC-1, apoptosis with Annexin V and PI, migration with transwell chambers, and tube formation with Matrigel.

**Results:**

ox-LDL decreased the nuclear Nrf2/Histone H3 to cytoplasmic Nrf2/GAPDH ratio, NQO1 mRNA, and protein levels. ox-LDL enhanced ROS production, induced the loss of membrane potential, and increased the cell shrinkage, pyknotic nuclei, and apoptosis of EPCs. Keap1 siRNA increased Nrf2 nuclear translocation, NQO1 mRNA transcription, and protein expression and prevented ROS generation and formation of JC-1 monomers. ox-LDL increased the activation of p38. SB203580 significantly eliminated ox-LDL induced inhibition of Nrf2 nuclear translocation, depression of NQO1 mRNA transcription, generation of ROS, and formation of JC-1 monomers in EPCs. Keap1 siRNA decreased the Bax/Bcl-2 ratio which was increased by ox-LDL in EPCs. ox-LDL decreased EPC migration and tube formation. Keap1 siRNA preserved the migration and tube formation of EPCs.

**Conclusion:**

ox-LDL activated EPCs p38/Keap1/Nrf2 pathway and induced oxidative stress, dysfunction, and apoptosis of EPCs.

## 1. Introduction

Coronary and cerebral artery occlusion induced by atherosclerotic plaque formation and disruption is one of the major causes of the mortality and morbidity within the developed and modern societies. Hypercholesterolemia induced damage of the vascular endothelial integrity is considered as the initial trigger of the development of atherosclerosis [1]. Lipid oxidation, especially oxidized-low density lipoprotein (ox-LDL), plays a vital role in the formation and progression of atherosclerosis. ox-LDL induces the development of atherosclerotic plaque primly through increases of oxidative stress and endothelial cells damage and reduced restoration of impaired endothelium in ischemic tissue [2].

Through homing to sites of endothelial damage, differentiating into mature endothelial cells, and incorporating into the endothelial lining, endothelial progenitor cells (EPCs) originated from hematopoietic stem cells are considered to play an important role in endothelial regeneration and vascular repair. It was reported that antioxidant proteins were much more highly expressed in EPCs than in endothelial cells [3]. However, recent studies showed that EPCs, unlike stem cells, were still vulnerable to environmental risk factors, particularly oxidative stress, which was abundant in the reparative ischemic environment [4].

Nuclear factor erythroid 2-related factor 2 (Nrf2) controls the expression of many genes encoding cellular redox regulators and antioxidant proteins by binding to antioxidant response element (ARE) [5]. Nrf2 plays a critical role in the regulation of redox balance, lipid metabolism, and foam cell formation in atherosclerosis [6]. Nrf2 has recently been taken as a new potential pharmacological target for the treatment of cardiovascular disease, diabetes, neurodegenerative diseases, cancer, and airway disorders [7]. Nrf2 activity is regulated by intracellular signals at multiple levels, including gene transcription, kinase-mediated phosphorylation, cytoplasm-nucleus trafficking, Kelch-like ECH-associated protein-1- (Keap1-) dependent and Keap1-independent proteasome degradation, and DNA binding [8]. Nevertheless, nuclear translocation is essential for activation of Nrf2 and subsequent antioxidant expression [9]. Under low oxidation conditions, Nrf2 is retained in the cytoplasm by combining to Keap1 and maintained at a low level by Keap1-dependent ubiquitination and proteasomal degradation system. In some cell types, ox-LDL has been reported to regulate Nrf2 activation and the expression of antioxidant and oxidative stress response genes [10]. However, whether ox-LDL influenced activation of Nrf2 and its related signals in EPCs has not been fully illustrated. In this work, we investigated the effects of ox-LDL on the level of activated Nrf2 in EPCs nuclear and the biological functions of EPCs, including migration and angiogenesis, as well as its relevance to oxidative stress and cell senescence. A preprint has previously been published [11].

## 2. Materials and Methods

### 2.1. Preparation of Human EPCs

The study was approved by the Institutional Review Board at General Hospital of Central Theater Command. EPCs were prepared as our previous report [12]. Peripheral Blood Mononuclear Cells were isolated from healthy human volunteers' peripheral blood sample. After isolation, total mononuclear cells (5 × 10^6^ cells/mL) were plated on culture dishes which were coated with human fibronectin (F0895; Sigma-Aldrich) and then maintained in endothelial basal medium (EBM, CC-3162; Lonza, Switzerland) which was supplemented with EGM Single Quots, 10 ng/mL VEGF (orb178366; Biorbyt, Missouri, USA) and 20% fetal bovine serum (FBS). The culture medium was replaced every 3 days. After 8 days of culture, adherent cells were labeled with DiI-acLDL (L3484, Invitrogen, Carlsbad, California, USA) and fluorescein isothiocyanate- (FITC-) labeled lectin from ulex europaeus lectin (L9006; Sigma-Aldrich; Merck KGaA). Cells which were double-positive of DiI-acLDL and FITC-labeled lectin were identified as EPCs, as reported previously.

### 2.2. ox-LDL Preparation

LDL which was separated from healthy volunteers' plasma after 12 hours of fasting was used to prepare ox-LDL. The protein content of LDL was determined by the modified Lowry method. LDL was incubated with CuSO4 (10 *μ*M) at 37°C for 24 hours, then dialyzed against a sterile solution of NaCl (150 mM), EDTA (1 mM), and polymyxin B (100 *μ*g/mL) (pH 7.4) twice. Agarose gel electrophoresis and generation of thiobarbituric acid were used to confirm the presence of ox-LDL.

### 2.3. Keap1 Silencing by siRNA

EPCs were transfected with siRNA using Lipofectamine™ 2000 (Invitrogen Ltd., Carlsbad, CA) transfection reagent according to the manufacturer's instructions. The target sequences of the Keap1-siRNA were as follows: sense strand—5′-GGAGUACAUCUACAUGCAU-3′, and antisense strand—5′-AUGCAUGUAGAUGUACUCC-3′, and scrambled siRNA control. After transfection of Keap1-siRNA for 6 hours, the culture medium was replaced, and then EPCs were incubated for another 24 hours to reach the silenced phase. The Keap1 knockdown efficiency was validated by Western blotting analysis.

### 2.4. Cytoplasmic and Nuclear Protein Extraction

EPCs grown to 80% confluency and were subjected to various treatments, then washed with ice-cold phosphate-buffered saline (PBS). The cytoplasmic protein and nuclear protein of EPCs were extracted using nuclear protein preparation kit and cytoplasmic protein preparation kit (P1200; BeiJing Applygen Technologies Inc., Beijing, China), respectively, according to the manufacturer's instructions. After gently scraping with 3 mL ice-cold PBS, EPCs grown on 10 cm dish were centrifuged at 600 g for 10 min at 4°C. Carefully aspirating the supernatant, EPCs were resuspended with 200 *μ*L ice cold CEB-A Mix and proteinase inhibitor, incubated for 15 min on ice to allow EPCs to swell, and then added 11 *μ*L ice-cold CEB-B. EPCs were vigorously vortexed for 10 s and centrifuged at 16, 000 g for 5 min at 4°C. The supernatant (cytoplasmic fraction) was carefully aspirated and stored at -80°C. The pellets were resuspended with 200 *μ*L ice-cold NEB Mix and proteinase inhibitor and vigorously vortexed. The suspension was placed on ice for 40 min and then centrifuged at 16, 000 g for 15 min at 4°C. The supernatants (nuclear extracts) were also stored aliquots at -80°C. Protein concentration of the supernatants was determined with the colorimetric assay (Bradford).

### 2.5. Western Blot Analysis

The cytoplasmic and nuclear protein samples were resolved in 12% sodium dodecyl sulfate polyacrylamide gel electrophoresis (SDS-PAGE) and electrotransferred onto nitrocellulose membrane (IPFL00010; Millipore). The membrane was incubated with primary antibodies at a dilution of 1 : 100 to 1 : 1000 at 4°C for 12 hours, washed out with TBS/T (Tris-buffered saline containing 0.2% Tween 20), exposed to HRP-conjugated anti-goat or anti-mouse secondary antibody (1 : 5000) for 1 hour, respectively, and then visualized by enhanced chemiluminescence detection reagents. Relative intensities of protein bands were analyzed with Image-Pro Plus 6.0 (Media Cybernetics, Silver Spring, MD, USA). Antibodies used in this study were as follows: anti-Nrf2 (Ab62352; Abcam), anti-Histone H3 (PAB33309; BIOSWAMP), anti-glyceraldehyde-3-phosphate dehydrogenase (GAPDH, FNab03345; Wuhan Fine Biotech Co., Ltd.), anti-NAD(P)H: quinone oxidoreductase 1 (NQO1) (Ab80588; Abcam), anti-Bax (FNab00810; Wuhan Fine Biotech Co., Ltd), anti-Bcl-2 (658701; BioLegend), anti-Keap1 (Ab139729; Abcam), anti-p-p38 (4511 T; Cell Signaling Technology), anti-p38 (622403; BioLegend), and anti-beta-actin (PAB36265; BIOSWAMP).

### 2.6. RT-PCR Analysis

The treated EPCs in each group were collected for the total RNA extraction using Trizol. The RT-PCR was performed with a total reaction volume of 10 *μ*L, including 10 ng of cDNA, 0.25 *μ*M forward and reverse primers, and 5 *μ*L SYBR-Green qPCR master mix. The amplified genes and the primers used were as follows: NQO1 (forward, CGCAGACCTTGTGATA and reverse, TGGCAGCGTAAGTGTA) and GAPDH (forward, ACAACTTTGGTATCGTGGAAGG and reverse, GCCATCACGCCACAGTTTC). The required number of cycles to generate a given threshold signal (Ct) was recorded for each sample. The mRNA transcription levels for each sample were analyzed using the 2-*ΔΔ*Cq.

### 2.7. Measurement of Intracellular Reactive Oxygen Species (ROS)

Intracellular ROS levels were detected using a fluorescence probe 2′,7′-dichlorofluorescin diacetate (DCFH-DA, D6883; Sigma-Aldrich, USA) as previously described [13]. After exposure to the indicated treatment, EPCs were incubated with 5 mM DCFH-DA at 37°C for 20 min in the dark and then washed three times with serum-free EGM-2 medium. The representative images of ROS generation were captured using a Leica DMIL LED-inverted fluorescence microscope (Leica, Germany). Samples without intervention were used as negative controls. ROS levels of treated groups were expressed as relative fluorescence intensity compared to the control group.

### 2.8. Analysis of Mitochondrial Transmembrane Potential (MMP)

MMP assay was performed using 5,5′,6,6′-tetrachloro-1,1′,3,3′-tetraethyl-imidacarbocyanine iodide (JC-1) whose aggregation reflect the inner mitochondrial membrane potential. When the MMP decreased, JC-1 selectively entered mitochondria and reversibly changed its color from red to green. After treatment, the EPCs were incubated with 10 *μ*g/mL JC-1 (T4069; Sigma-Aldrich, USA) at 37°C for 15 min and then washed with serum-free EGM-2 medium three times. The fluorescence color was monitored using a Leica DMIL LED-inverted fluorescence microscope (Leica, Germany). Analyzed from six random fields with the Image-Pro Plus 6.0 (Media Cybernetics, Silver Spring, MD, USA), the *ΔΨ*m in each group was calculated as the ratio of cells with green fluorescence to cells with red plus green fluorescence.

### 2.9. Apoptosis Assay

Apoptosis of EPCs was quantified using an Annexin V-FITC/propidium iodide (PI) Apoptosis Kit (40302ES20; Yeasen, USA) following the manufacturer's instruction. After intervention, EPCs were harvested and washed and then suspended in 1× Binding buffer and 100 *μ*L of cell suspension which was mixed with 5 *μ*L of PI and 5 *μ*L of Annexin V-FITC. After incubation for 15 min in the dark, the apoptosis of EPCs was measured using a flow cytometer (CytoFLEX S, Beckman, USA).

In addition, morphological changes of the nuclei of apoptotic EPCs were quantified by 4′,6-diamidino-2-phenylindole (DAPI, D9542; Sigma-Aldrich, USA) staining. After treatment, 100 ng/mL DAPI was added into the culture media and incubated at 37°C for 20 min. After DAPI-staining, viable cells displayed normal nuclear size and uniform nuclear fluorescence, whereas apoptotic EPCs showed condensed, fractured, or distorted nuclei. The pyknotic nuclei were manually counted in six random fields in each well and recorded as the percentage of the total EPCs nuclei number.

### 2.10. Migration Assay of EPCs

EPC migration was identified by transwell chemotaxis assay with Boyden chamber. In each 24-well plate, 1 × 10^4^ EPCs were plated on the top side of the polycarbonate transwell filter (8 *μ*m pore size, MCEP24H48; Millipore, Massachusetts, USA). EBM-2 medium containing 10% FBS was added into the lower chamber. Then, the growth medium was replaced with fresh EGM containing ox-LDL or Keap1 siRNA. After 24 hours of culture, the nonmigrative cells in the upper chambers were gently scrubbed out and the cells on the bottom of the membrane surface were fixed with 4% paraformaldehyde, stained with 0.5% crystal violet solution (PAB180004; Bioswamp, Wuhan, China), and then counted under microscope (Leica DMIL LED, Leica Company, Germany). The number of migrated EPCs was determined from 5 random 100x fields for each group, and each experiment was performed in triplicate.

### 2.11. EPC Tube Formation Assay

The tube formation assay was performed using Matrigel (356237; CORNING, Germany). Matrigel was dissolved at 4°C, with 100 *μ*L added into 48-well plates per well, and then incubated for 30 min at 37°C. After intervention, 2 × 10^4^ EPCs per well were seeded onto the Matrigel and cultured at 37°C for 4 hours in the presence of 5% CO_2_. Images of capillary-like structures were captured under a microscope at 5 random 100x magnification fields for each group. The average numbers of branch point that indicated the formation of tube were counted and compared using Image-Pro Plus 6.0 (Media Cybernetics, Silver Spring, MD, USA).

### 2.12. Statistical Analysis

All data were expressed as the mean ± SEM or percentage. Statistical analysis between multiple groups was made with one-way ANOVA, and comparisons between two groups were performed using unpaired Student's *t*-test. *p* < 0.05 was considered statistical significance.

## 3. Results

### 3.1. EPC Morphology and Identification

**Figure 1:**
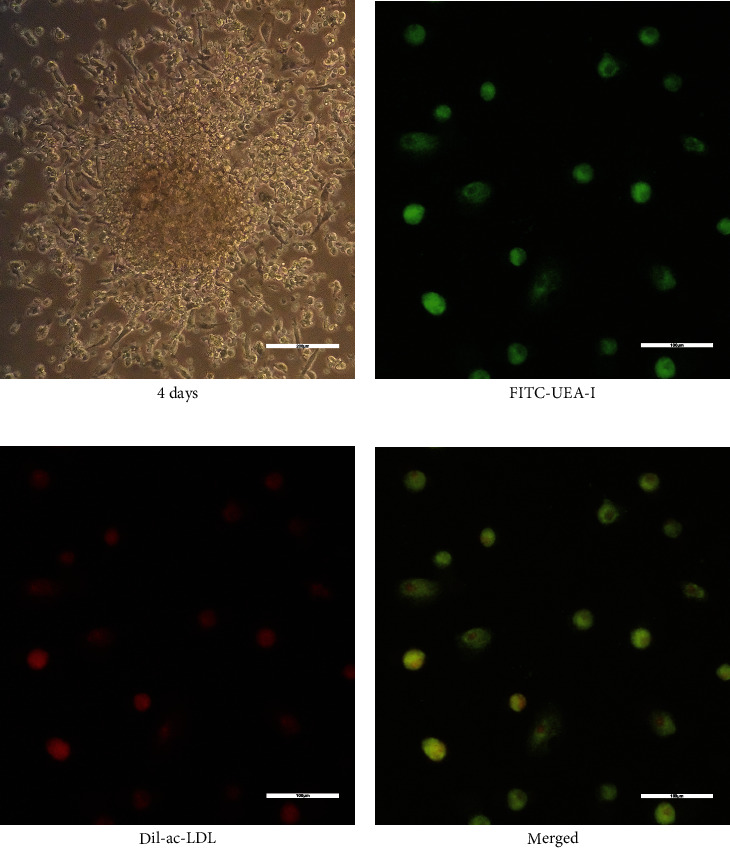
Characterization of endothelial progenitor cells (EPCs) from human peripheral blood. (a) The formation of EPC colonies. Cord-like structures formed by adherent cells. Spindle-shaped cells sprouted from the edges of cell clusters. Scale: 200 *μ*m. (b–d) The fluorescent image showed Dil-acetylated low-density lipoprotein (Dil-acLDL) incorporation (red) and FITC lectin (FITC-UEA-1) binding (green) by EPCs. Scale: 100 *μ*m.

After 24 hours of culture in EBM, cells began to adhere to plate wall. Colonies were observed at 48 hours, and most of the colonies appeared at 4 days. After 8 days of culture, spindle-shaped or cobblestone-like adherent cells were observed. Most of these adherent cells uptook Dil-ac-LDL and were positively stained with UEA-I (Figures 1(a)–1(d)).

### 3.2. ox-LDL Inhibited Nrf2 Nuclear Translocation

**Figure 2:**
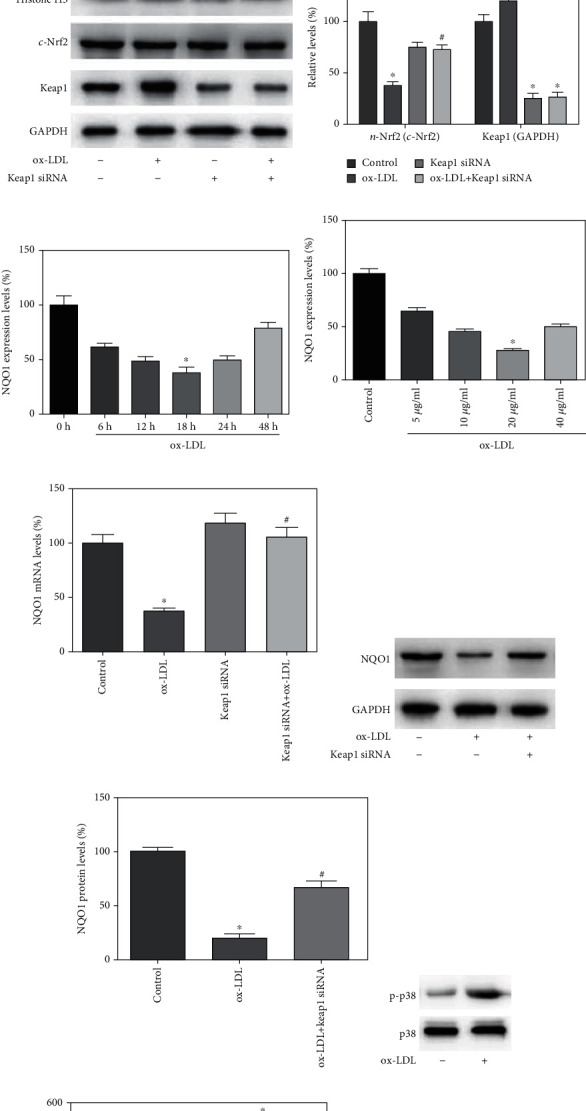
ox-LDL inhibited Nrf2 nuclear translocation via MAPK-p38 pathway. (a, b) EPCs were exposed to different concentration of ox-LDL (5-20 *μ*g/mL) for 24hours. The ratio of the control group was assigned a value of 1. Quantification of relative quantity of n-Nrf2/Histone H3 and c-Nrf2/GAPDH showed that ox-LDL concentration dependently decreased n-Nrf2/Histone H3, but not significantly influenced the c-Nrf2/GAPDH levels. (c, d) EPCs were transfected with specific siRNA against Keap1 or silencer select negative control (control group) for 24 hours. Western blot detection showed that Keap1 expression level was significantly inhibited by Keap1 siRNA transfection. (e, f) The inhibition of Nrf2 nuclear translocation in response to ox-LDL was reversed by pretreatment with the Keap1 siRNA, as shown by Western blot. (g, h) ox-LDL time- and dose-dependently decreased NQO1 mRNA transcription. (i) Pretreatment with Keap1 siRNA reversed ox-LDL-induced downregulation of NQO1 mRNA transcription. (j, k) Pretreatment with Keap1 siRNA reversed ox-LDL-induced downregulation of NQO1 protein expression. (l, m) ox-LDL significantly increased p38 phosphorylation. (n, o) Treatment with 1 *μ*M SB203580, a specific p38 inhibitor, reversed the ox-LDL-induced downregulation of Nrf2 nuclear translocation. (p) Pretreatment with SB203580 reversed ox-LDL-induced downregulation of NQO1 mRNA transcription. ^∗^*p* < 0.01 vs. control. ^#^*p* < 0.01 vs. ox-LDL group.

To investigate the effect of ox-LDL on Nrf2 nuclear translocation in EPCs, the cytoplasmic Nrf2 (c-Nrf2) level and nuclear Nrf2 (n- Nrf2) level in EPCs after treatment of different concentration of ox-LDL were determined by Western blot. The nuclear levels of Nrf2 were decreased to 21.9% by 20 *μ*g/mL ox-LDL treatment for 12 hours (Figures 2(a) and 2(b)). Analysis of n-Nrf2 and c-Nrf2 could further ascertain the Nrf2 location and thereby identify the influence of ox-LDL on Nrf2 preservation and nuclear translocation. Comparison of the n-Nrf2/Histone H3 to c-Nrf2/GAPDH ratio among all experimental groups showed that 10 *μ*g/mL and 20 *μ*g/mL ox-LDL, respectively, caused a decrease of the Nrf2 nuclear level by 66.1% and 71.9% (*p* < 0.05, Figure 2(b)), with similar c-Nrf2 levels. However, the mRNA levels of Nrf2 were not influenced by ox-LDL treatment, indicating that ox-LDL deceased n-Nrf2 level probably by the augmentation of the lysine acetylation and ubiquitination of Nrf2. To assess whether Keap1-dependent or Keap1-independent proteasome degradation of Nrf2 was implicated in ox-LDL induced Nrf2 downregulation, knockdown of Keap1 by the transfection of Keap1 siRNA was performed. The Keap1 protein levels were efficiently depleted by about 70% after 24 hours of treatment of Keap1-siRNA as evidenced by Western blot assay (Figures 2(c) and 2(d)). Compared to the ox-LDL-treated group, the nuclear level of Nrf2 was substantially increased in the Keap1 knockdown plus ox-LDL treatment group (Figures 2(e) and 2(f)). These results suggested that ox-LDL might decrease Nrf2 nuclear level by Keap1-dependent Nrf2 degradation and inhibition of nuclear translocation.

### 3.3. Nrf2 Mediated ox-LDL-Induced NQO1 Depression in EPCs

NQO1 a typical direct target gene of Nrf2 exerts a protective role in alleviating EPCs damage [14]. To figure out the effects of ox-LDL on the expression of Nrf2 targeted genes in EPCs, we characterized concentration- and time-dependent regulation of NQO1 mRNA by ox-LDL in EPCs through RT-PCR. Results showed that after 10 *μ*g/mL ox-LDL treatment, NQO1 transcription levels sharply decreased to 67.9% at 6 hours, reached a minimum of 39.4% at 18 hours, and returned to control levels by 48 hours and later (Figure 2(g)). Consistently, NQO1 transcription levels attained a minimal level after 20 *μ*g/mL ox-LDL treatment for 18 hours (Figure 2(h)). Additionally, pretreatment of EPCs with Keap1 siRNA 24 hours before ox-LDL treatment resulted in a significant increase of NQO1 transcription compared to those without Keap1 siRNA (Figure 2(i)).

To determine whether ox-LDL influenced NQO1 protein expression level, we performed immunoblotting analysis of NQO1 protein. In EPCs challenged with 20 *μ*g/mL ox-LDL for 24 hours, the NQO1 protein level was markedly decreased, and pretreatment of EPCs with Keap1 siRNA transfection 24 hours before ox-LDL treatment significantly promoted NQO1 levels (Figures 2(j) and 2(k)). These results indicated that ox-LDL reduced NQO1 levels at the transcription and protein level in a time- and concentration-dependent manner in EPCs, which might have been implicated to Keap1-dependent regulation of Nrf2.

### 3.4. ox-LDL Inhibited Nrf2 Activation via p38 Signal

Previous studies showed that redox in EPCs was regulated by several signal transduction pathways. The mitogen-activated protein kinase (MAPK) p38 recently identified as a modulator of the proliferation of ex vivo progenitor cells plays a primordial role in response to the changes of the cellular redox [15]. In various human cell lines, MAPK p38 was reported to suppress Nrf2 activation via the promotion of the stabilization of the interaction between Keap1 and Nrf2 and the increase of Nrf2 breakdown [16]. To elucidate the molecular mechanisms underlying the inhibition of Nrf2/NQO1 by ox-LDL, we evaluated the effect of ox-LDL on the phosphorylation of MAPK p38 in EPCs with Western blot. Results showed that 20 *μ*g/mL ox-LDL treatment for 2 hours significantly increased the activation of p38 (Figures 2(l) and 2(m)). To further understand the role of p38 MAPK in EPCs redox regulation, we investigated the impact of p38 MAPK on Nrf2 nuclear translocation and NQO1 expression in EPCs, using a specific p38 MAPK inhibitor SB203580. Results showed that 1 *μ*M SB203580 pretreatment significantly eliminated ox-LDL-induced inhibition of the Nrf2 nuclear translocation (Figures 2(n) and 2(o)) and the NQO1 mRNA transcription levels in EPCs (Figure 2(p)). However, the mRNA and protein level of Keap1 has not been influenced by SB203580. These results suggested that ox-LDL inhibited the Keap1/Nrf2 antioxidative defense pathway probably via activating p38 signaling pathway.

### 3.5. Nrf2 Mediated ox-LDL-Induced EPC Oxidative Stress and Apoptosis

**Figure 3:**
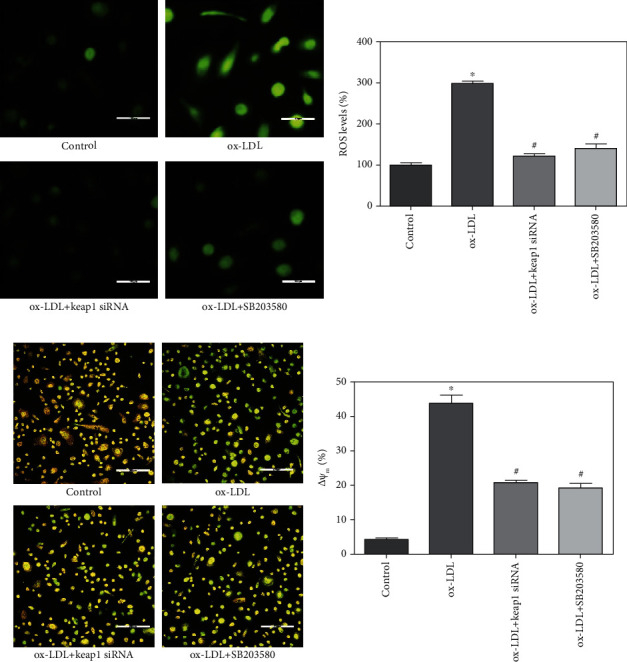
Nrf2 mediated ox-LDL-induced EPC oxidative stress and mitochondrial dysfunction. After being pretreated with Keap1 siRNA or 1 *μ*M SB203580, EPCs were exposed to ox-LDL (20 *μ*g/mL) for 6 hours. The control well was treated with medium alone. Intracellular ROS levels were estimated using the probe DCFH-DA. (a) Representative images of EPCs treated by control, 20 *μ*g/mL ox-LDL, 20 *μ*g/mL ox-LDL plus Keap1 siRNA, and 20 *μ*g/mL ox-LDL plus 1 *μ*M SB203580. Scale: 100 *μ*m. (b) Fluorescence was read at 520 nm for emission and 485 nm for excitation. Quantification of relative fluorescent intensity showed that ox-LDL-induced elevation of ROS levels in EPCs was reversed by pretreatment of Keap1 siRNA and SB203580. (c) Represent images of EPCs stained by JC-1 in each group after the treatment. Scale: 200 *μ*m. (d) ox-LDL-induced EPC mitochondrial dysfunction which was reversed by Keap1 siRNA. ^∗^*p* < 0.01 vs. control. ^#^*p* < 0.01 vs. ox-LDL group.

To determine the role of Nrf2 and p38 MAPK in ox-LDL-induced oxidative stress in EPCs, the ROS generation was assessed by detecting the oxidation with DCFH-DA under fluorescent microscopy after pretreatment of EPCs with Keap1 siRNA and SB203580 before ox-LDL treatment. DCFH-DA staining showed that the treatment of EPCs with ox-LDL for 6 hours significantly enhanced ROS production in EPCs. However, the pretreatment with Keap1 siRNA or SB203580 both significantly prevented ox-LDL induced ROS generation in EPCs. Pretreatment of EPCs with Keap1 siRNA or SB203580 significantly reduced the ROS levels in EPCs (reduced from 301.2% to 121.6% and 142.1%, respectively, comparing to the ox-LDL group, *p* < 0.01, Figures 3(a) and 3(b)). These results indicated that the increase of ROS production in EPCs after ox-LDL stimulation primarily attributed to the inactivation of Nrf2 and activation of p38.

One of the early critical events in cell apoptosis is the loss/disruption of MMP, which eventually causes the initiation and activation of apoptotic cascades [17]. The effects of ox-LDL on EPCs MMP were evaluated with JC-1 staining under a fluorescence microscope. Treatment of EPCs with 20 *μ*g/mL ox-LDL significantly increased the formation of monomeric JC-1 with green fluorescence in mitochondria, indicative of a loss of membrane potential (Figure 3(c)). Keap1 siRNA and SB203580 pretreatment decreased the formation of JC-1 monomers (Figure 3(d)), suggesting that activation of Nrf2 and inhibition of p38 protected EPCs from apoptosis.

**Figure 4:**
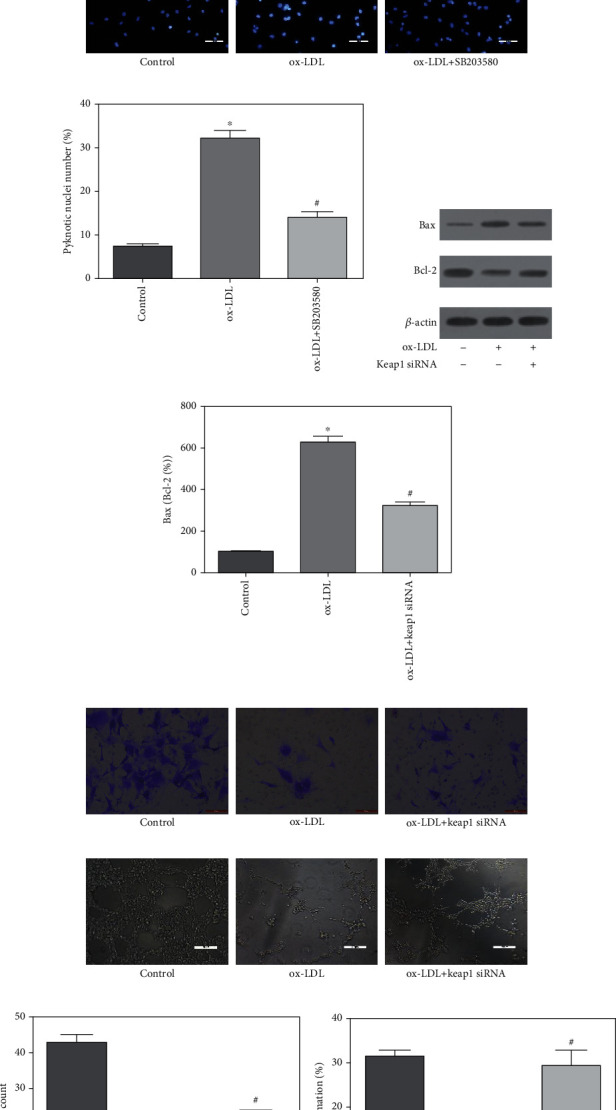
ox-LDL impaired EPC migration and tube formation and induced apoptosis via Nrf2 or p38 pathway. EPCs were pretreated with Keap1 siRNA or 1 *μ*M SB203580 and then exposed to ox-LDL (20 *μ*g/mL) for 6 hours. (a) Apoptosis was determined through Annexin V-FITC and PI double-staining using flow cytometry after ox-LDL or 1 *μ*M SB203580 treatment for 6 hours. ox-LDL increased the apoptotic rate of EPCs. Pretreatment of SB203580 reduced the apoptotic rate of EPCs. (b, c) EPC apoptosis was quantified by manually counting pyknotic nuclei after DAPI (Sigma-Aldrich) staining. ox-LDL increased the apoptotic rate of EPCs. Pretreatment of SB203580 reduced the apoptotic rate of EPCs. Scale: 100 *μ*m. (d) Represented Western blot images of the protein expressions of Bax and Bcl-2 in EPCs after treatment of ox-LDL or Keap1 siRNA. (e) Quantification of relative quantity of Bax and Bcl-2 showed that ox-LDL significantly increased Bax/Bcl-2 ratio, which was blocked by the pretreatment of Keap1 siRNA transfection. Migration of EPCs was examined by transwell chemotaxis assay. (f) The representative images of migrated EPCs in the control group, ox-LDL group, and Keap1 siRNA pretreatment plus the ox-LDL group (magnification: ×100). Scale: 50 *μ*m. (g) The presentative photomicrographs of tube formation of EPCs under the treatment of ox-LDL and Keap1 siRNA pretreatment. Scale: 200 *μ*m. (h) Quantitative data show that ox-LDL significantly reduced the numbers of migrated EPCs, but Keap1 siRNA significantly increased the numbers of migrated cells. Values are the means ± SD from 3 independent experiments. The angiogenic function of EPCs under exposure to ox-LDL or Keap1 siRNA was determined by tube formation assay. (i) Quantification analysis of the number of tube branches showed that Keap1 siRNA pretreatment ameliorated EPC angiogenic function which were inhibited by ox-LDL. ^∗^*p* < 0.01 vs. control. ^#^*p* < 0.01 vs. ox-LDL group.

The effects of ox-LDL on EPCs survival were examined with both manually counting DAPI stained pyknotic nuclei and flow cytometric analysis of Annexin V and PI. The Annexin V/PI double staining showed that treatment of EPCs with 20 *μ*g/mL ox-LDL for 6 hours increased the apoptotic proportion from 9.13% to 44.30% (Figure 4(a)), concomitant with an increase in cell shrinkage and pyknotic nuclei (Figures 4(b) and 4(c)). However, pretreatment with SB203580 decreased ox-LDL-induced EPC apoptotic proportion to 22.03% (Figures 4(a)–4(c)).

To determine whether the apoptotic pathway was implicated to ox-LDL-induced EPC apoptosis, the expression of proapoptosis factor, Bax, and prosurvival factor, Bcl-2, was examined with Western blot. As shown in Figures 4(a) and Figure 4(b), ox-LDL increased 2-folds of the Bax expression and simultaneously decreased 30% of the Bcl-2 expression, eventually leading to an approximate 4-fold increase of Bax/Bcl-2 ratio in EPCs, whereas these effects were blocked by the pretreatment with Keap1 siRNA transfection (Figures 4(d) and 4(e)). All these results suggested that activation of Nrf2 significantly inhibited ox-LDL-induced ROS production, mitochondria membrane potential reduction, apoptotic pathway activation, and cell apoptosis.

### 3.6. ox-LDL Inhibited EPC Migration and Tube Formation via Nrf2 Pathway

The migration activity of EPCs after ox-LDL stimulation was detected using transwell chamber assay. The transwell chambers were incubated in a 37°C incubator for 24 hours. The migrated EPCs were stained with 1% crystal violet solution and counted in 5 random high-power (100 ×) microscope fields. Results showed that treatment with ox-LDL significantly reduced the migration ability of EPCs (Figure 4(f)). To test the role of Nrf2 signal in the loss of EPC migration activity, Keap1 siRNA was pretreated 0.5 hours before addition of ox-LDL to EPC culture media. Results showed that Keap1 siRNA preserved the migration capacity of EPCs (Figure 4(h)).

The present study investigated the effect of ox-LDL and Keap1 siRNA on the angiogenesis of EPCs by performing a tube formation assay. The formation of tube-like structures was decreased in ox-LDL-treated EPCs, whereas pretreatment with Keap1 siRNA significantly increased the tube formation of EPCs (Figures 4(g) and 4(i)). The results suggested that activation of p38 attenuated the inhibition of ox-LDL on EPC tube formation.

## 4. Discussion

Atherosclerosis resulting in many vascular diseases is thought to be initiated and promoted by vascular endothelial injury and oxidative stress [18]. Endothelial cells usually work as the first defensive line in vessels against harmful substances in the blood. However, when vascular endothelial cells are exposed to excessive external stimulus, their structure and function become impaired [19]. EPCs with the inherent capacity to differentiate into mature endothelial cells play an important role in postischemic vascular repair [20]. ox-LDL which accumulates in the vascular wall promotes the formation and development of atherosclerotic plaques by inducing vascular endothelial apoptosis and impairment of EPC function [21]. It was reported that the antioxidant genes were much more highly expressed in EPCs than that in endothelial cells [22]. Nevertheless, in several pathological conditions, the overproduction of ROS in EPCs induces oxidative stress, promotes lipid peroxidation, and then results in EPC senescence and dysfunction [23]. However, in the present study, we found that ox-LDL increased EPC ROS production, mitochondrial dysfunction, and apoptosis. Mitochondrial respiratory chain is considered to be the principal source of ROS in the cells [24]. Generally speaking, the leaked electrons from mitochondrial complexes partially reduce oxygen to O_2_^−^ and convert about 1-2% of the total rate of oxygen consumption into ROS [25]. The redox homeostasis in cells is depended on the balance between the production of ROS from mitochondria and the expression of antioxidative genes. In the present study, ox-LDL induced oxidative stress of EPCs probably via both mitochondria dysfunction and inhibition of antioxidative system. Furthermore, the impaired EPCs influence the balance between the damage and the repair of endothelial cells and accelerate formation of atherosclerosis. These results were consistent with the previous reports in which ox-LDL exerted a deleterious effect on ability and survival of EPCs via ROS generation and dysfunction of antioxidant enzymes [26].

Nrf2 is ubiquitously expressed in cells and closely associated with atherosclerotic pathogenesis [27]. In endothelial cells, activation of Nrf2 was reported to ameliorate survival, proliferation, and angiogenic function of endothelial cells in vivo and in vitro [28]. The activation of Nrf2 is now widely considered to be controlled by Keap1 which acts as an intracellular sensor for endogenous and exogenous electrophiles and oxidants [29]. In the condition of oxidative stress, Nrf2 releases from Keap1 in the cytoplasm, translocates into the nucleus, binds to ARE, and induces the expression of antioxidant genes, such as NQO1 and HO-1 [30]. Many studies showed that the increase of these antioxidant genes was synchronous after activation of Nrf2 pathway [31]. However, HO-1 which reduces oxidative stress via reduction of heme in cells [32] probably is not the principal antioxidative pathway in EPCs, because of the low production of heme in EPCs. NQO1 as a cytosolic oxidoreductase plays a critical role in regulating the ROS levels. NQO1 catalyzes the oxidation of NAD(P)H to NAD(P)+ and depresses the ROS levels through inhibition of quinones from entering the one electron reduction to ROS and semiquinone free radicals [33]. So this powerful antioxidant protein eliminates accumulated ROS quickly and maintains cell redox balance. In the present study, we found that ox-LDL reduced the nuclear translocation of Nrf2 and also NQO1 expression at the transcriptional and protein levels in EPCs in a time- and concentration-dependent manner. These results were consistent with the previous report in which ox-LDL induced endothelial dysfunction via the depression of antioxidant protein [34]. However, we also found that ox-LDL did not influence the Nrf2 mRNA transcription levels, suggesting that ox-LDL probably inhibited Nrf2 not through inhibition of Nrf2 mRNA transcription. To further define the role of Keap1 in the regulation of Nrf2 activity, we employed Keap1 siRNA to regulate Nrf2 signal. The results showed that Keap1 siRNA increased NQO1 mRNA transcription and protein expression levels. It suggested that Keap1-dependent degradation of Nrf2 and inhibition of Nrf2 nuclear translocation participated into the inhibition of Nrf2/ARE system by ox-LDL. However, in physiological conditions, for the high expression of antioxidant gene and the very low production of ROS in EPCs, the ROS-driven dissociation of Nrf2 from Keap1 might not be the dominant pathway that regulates the activity of Nrf2 in EPCs.

The MAPK p38 signal pathway which is activated by many extracellular physiological stimuli and environmental factors plays an important role in the pathogenesis of atherosclerosis [35]. MAPK p38 signal was reported to induce endothelial cell apoptosis and inflammation [36]. It has been reported that ox-LDL elicited the increases of p38 MAPK phosphorylation and tremendous ROS generation in human umbilical vein endothelial cells [37], whereas previous studies showed that p38 MAPK inhibited the Keap1/Nrf2/ARE pathway via the regulation of Nrf2 stability [38]. In the present study, we found that ox-LDL significantly activated p38 and the inhibition of p38 MAPK reversed the depression of Nrf2 nuclear translocation and NQO1 mRNA transcription by ox-LDL in EPCs. These results suggested that ox-LDL inhibited the Keap1/Nrf2 antioxidant defense pathway probably via activating p38 signaling pathway. The present study also found that in EPCs the inhibition of p38 MAPK significantly reversed ox-LDL-induced ROS generation, mitochondrial dysfunction, and apoptosis. The results suggested that the p38/Keap1/Nrf2 pathway played a central role in control of ox-LDL-induced EPC oxidative stress and apoptosis. The p38/Keap1/Nrf2 pathway could be considered to be a different pathway in the ox-LDL regulated Nrf2 activity in these kinds of high antioxidant gene expression cells. It explained why the antioxidative defensive system did not worked when ox-LDL induced overproduction of ROS in EPCs.

Several studies showed that the migration and tube formation of EPCs were impaired in many pathological conditions [39]. Oxidative stress has always been implicated in mediating the apoptosis and dysfunction of EPCs [40]. Previous studies showed that ox-LDL-induced EPC oxidative stress via the activation of specific receptor and proinflammatory factor [41]. Inflammation pathway and oxidative stress are two important factors in the regulation of EPC function and fate [42]. The participation of p38 pathway and ROS in inflammation response has been both reported in many studies before [43]. It could be presumed that in EPCs after ox-LDL treatment, the elevated inflammation might increase the ROS production and apoptosis. However, these indirect effects should not be the principle pathway through which ox-LDL induced EPC oxidative stress, for its long conduction process, and also has not been tested in the present study. Theoretically, the ox-LDL-induced EPC oxidative stress could be counterworked by activation of antioxidant defense system. But in the present study, we found that ox-LDL inhibited antioxidant signal Nrf2/Keap1 and that the upregulation of Nrf2 through the Keap1 knockdown decreased the EPC ROS generation and mitochondrial dysfunction and ameliorated the migration ability and tube formation of EPCs. These results implicated that ox-LDL changed the equalization between the oxidative damage and the activity of antioxidant defense system and furthermore induced accumulation of ROS and apoptosis of EPCs. These results were consistent with previous reports in which lack of Nrf2 attenuated survival, proliferation, and angiogenic function of EPCs [44].

Bax and Bcl-2 have been identified to be involved in the control or execution of apoptosis [45]. Bax permeabilizes the outer membrane of mitochondria and succeeds the release of proapoptotic molecule cytochrome c. Cytosolic cytochrome c binds with many proapoptotic factors to create a protein complex known as apoptosome. However, effects of Bax could been inhibited by Bcl-2, so the Bax/Bcl-2 ratios have been implicated in the mitochondrial dysfunction induced intrinsic apoptotic pathway [46]. Moreover, the increase of bax/Bcl-2 ratios leads to the activation of caspase-3 and thus determines a cell's susceptibility to undergo apoptosis [47]. In the apoptotic cells, caspase-3 could be activated by both intrinsic (mitochondrial) and extrinsic (death ligand) pathways [48]. The present study showed that ox-LDL increased Bax expression and decreased Bcl-2 expression, resulting in a significant increases of Bax/Bcl-2 ratio within EPCs. In the present study, the apoptosis of EPCs has been testified by flow cytometric analysis of Annexin V and PI, and the mechanisms of ox-LDL-induced EPC apoptosis were shown to be the activation of the intrinsic apoptotic pathway Bax/Bcl-2. These effects were partially blocked by the Keap1 knockdown. These results suggested that upregulation of Nrf2 signal inhibited the activation of a proapoptotic signal pathway which was induced by ox-LDL.

## 5. Conclusions

All these results suggested that ox-LDL induced EPC oxidative stress by Keap1 dependent Nrf2 degradation and inhibition of its nuclear translocation. Treatment of ox-LDL inhibited the Keap1/Nrf2 antioxidant defense pathway probably via activating p38 signaling pathway. The p38/Keap1/Nrf2 pathway played a central role in ox-LDL-induced ROS production, mitochondria membrane potential reduction and cell apoptosis, and activation of the apoptotic pathway Bax/Bcl-2.

## Data Availability

The data used to support the findings of this study are included within the article.
